# Clinical importance of serum secreted clusterin in predicting invasive breast cancer and treatment responses

**DOI:** 10.1080/21655979.2020.1868732

**Published:** 2021-01-28

**Authors:** Qing-Feng Chen, Lei Chang, Qun Su, Ying Zhao, Bin Kong

**Affiliations:** a Department of Breast Surgery, The Affiliated Hospital of Qingdao University, Qingdao, China; b Department of Clinical Lab, The Affiliated Hospital of Guanxi Medical University, Nanning, China

**Keywords:** Invasive breast cancer, diagnosis, prognosis, chemotherapy, clusterin

## Abstract

Enhanced serum secreted clusterin (sCLU) protein was associated with progression, poor prognosis and chemotherapy sensitivity evaluation in malignant patients. However, the clinical significance of serum sCLU protein levels in patients with invasive breast cancer (IBC) is unknown. In this study, the serum sCLU protein in 2648 patients with IBC was detected. The diagnostic value and treatment responses of serum sCLU protein in patients with IBC were also performed. The results showed that the serum sCLU protein level was signiﬁcantly higher in IBC patients compared to the healthy controls (P < 0.0001), and strongly correlated with higher clinical tumor stage (P < 0.001), lymph node metastasis (P < 0.001), shorter overall survival (OS) (P = 0.032) and disease-free survival (DFS) (P = 0.029), respectively. Using the cutoff value of 18.46 μg/mL, the sensitivity and speciﬁcity were 86.26% and 73.46% to separate IBC patients from noncancerous and healthy controls. The postoperative patients showed lower serum sCLU levels compared to the preoperative patients (P = 0.003). The chemoresistant patients showed higher serum sCLU levels compared to the chemosensitive patients (P < 0.001). These data indicated that serum sCLU levels are effective indicators for diagnosis and chemotherapy sensitivity evaluation in patients with IBC.

## Graphical abstract

**Figure:**
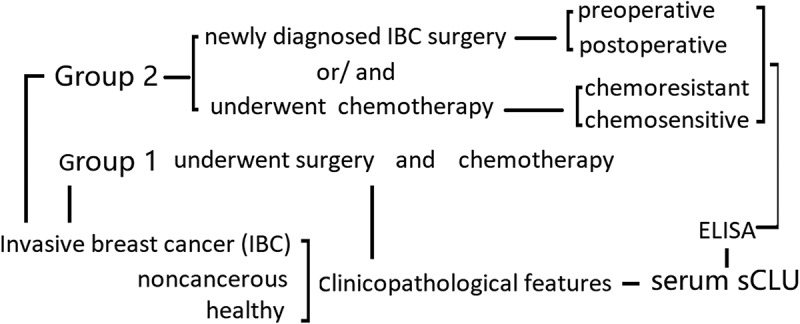

## Introduction

Breast cancer is the most common cancer in Chinese women, with 12.2% of all newly diagnosed breast cancers and 9.6% of all deaths from breast cancer worldwide [1]. With the improvement of early diagnosis technology of breast cancer, 5–10% of patients still have distant metastases at the time of diagnosis [2]. Standard chemotherapy and hormonal therapy options can not significantly reduce recurrence and metastasis, which results in 90% of the deaths in breast cancer patients [2–4]. Numerous prognostic factors are frequently used for making clinical decisions in breast cancer [5,6], but they are not routinely used in clinical practice because of their certain limitations and defect [7,8]. Therefore, it is urgent to find highly sensitive and specific molecular markers for diagnosis, prognostic judgment and evaluation of treatment response in patients with breast cancer.

Epidermal growth factor receptor (EGFR) serves as a co-target for dual/pan-EGFR-inhibitors in breast cancer [9]. Current evidence indicates associations between low baseline serum-EGFR and shorter survival or reduced response to treatment in patients with advanced breast cancer, especially in patients with estrogen and/or progesterone receptor-positive tumors. However, the prognostic and predictive value of EGFR and EGFR-ligands in blood has only been investigated in highly selected subsets of breast cancer patients and most studies were small [9–11]. The non-coding RNA sequences have been shown to be dysregulated in breast cancer. Despite showing immense promise, miRNAs have not been successfully implemented in the clinical setting due to a lack of a standardized approach which has resulted in conflicting results [12]. However, further studies are required to specifically isolate cancer exosomes from patient plasma and establish exosome microRNA signatures indicative of breast cancer. Following a diagnosis of breast cancer, measurement of serum CA 15–3 or CEA or uPA/PAI-1 and Oncotype DX may be used for determining prognosis and identifying lymph node-negative patients who may be spared from having to receive adjuvant chemotherapy [13]. However, the sensitivity and specificity of these biomarkers are relatively low for breast cancer.

Clusterin (CLU), a highly conserved 78–80 kDa heterodimeric glycoprotein, widely exists in a variety of tissues, cells and body fluids. CLU exists in at least two sets of isoforms: The secreted forms (sCLU) (∼75-80 kDa) and non-secreted nuclear forms (nCLU) (∼45-60 kDa). sCLU exhibits pro-survival functions [14–17] and nCLU appears to be pro-apoptotic functions [18,19]. sCLU is overexpressed in many types of malignant tumors, and inforced sCLU expression could promote carcinogenesis and tumor progression [20–23]. In addition, sCLU was also used as a marker to evaluate diagnosis and metastasis potential in many malignant tumors [24–27]. Previous study showed that sCLU was overexpressed in osteosarcoma (OS), and higher sCLU expression was related to metastasis and chemoresistance [28]. Higher preoperative sCLU expression in breast cancer was associated with CAF resistance and TNF-alpha-induced apoptosis in breast cancer cells in vitro [29,30]. Furthermore, targeting sCLU inhibited tumor growth and metastasis in breast cancer cells *in vivo* [31]. In this study, we evaluated whether serum sCLU levels could be used as a biomarker for the diagnosis and evaluating treatment responses in breast cancer patients with higher sensitivity and speciﬁcity.

## Materials and Methods

### Patients and serum samples

The patients with invasive breast cancer (IBC), noncancerous patients and healthy controls were collected between Jan 2012 and March 2014. The patients were divided into two groups: the first group was the control group and the IBC patients that underwent surgery or chemotherapy. Among them,1998 patients with pathologically confirmed IBC, 180 patients with pathologically confirmed noncancerous breast lesions and 170 healthy controls. The clinicopathological parameters were evaluated. The patients without pathologic diagnosis or with multiple cancers, or with adjuvant radiotherapy, or lost after operation were not included in the study. Plasma samples were collected and stored at −20°C from all participants. The clinicopathologic characteristics of the IBC patients were summarized in (Table 1).

**Table 1.** Clinicopathologic characteristics of patients with invasive breast cancer

Characteristic	Static group (n = 1998)	Dynamic group (n = 650)	
		Surgery (n = 160)	Chemotherapy (n = 490)
Age (years)			
<50	923	73	178
≥50	1075	87	312
Tumor size (Diameter)			
≤ 2 cm	288	18	36
>2 cm≤5 cm	1350	106	370
>5 cm	360	36	84
Histopathological Grades			
G1	230	19	61
G2	1478	121	348
G3	290	20	81
Clinical Stages			
I	208	14	41
II	970	80	231
III	820	66	218
Lymph metastasis			
Yes	1180	94	316
No	818	66	174
Distant metastasis			
Yes	115	19	64
No	1883	141	426
Subtype			
Luminal A	320	21	59
Luminal B	1111	92	293
HER2 (+)	211	16	48
Triple negative	356	31	90
Menopause			
Yes	798	70	216
No	1200	90	274
ER			
Positive(+)	1313	102	322
Negative(-)	685	58	168
PR			
Positive(+)	1017	91	286
Negative(-)	981	69	204

sThe second groups were the newly diagnosed IBC patients (n = 650), of which 160 patients received surgery and 490 patients underwent chemotherapy. For surgical patients, serum samples were collected 24 h before surgery, 48 h-72 h days after surgery, respectively. For chemotherapy patients, the baseline serum samples were collected before the ﬁrst chemotherapy cycle and subsequent serum samples in any treatment cycle. All patients follow the standard chemotherapy regimen. Two milliliters of whole blood was collected and centrifuged and then stored at −20°C. This study was approved by the affiliated Hospital of Qingdao University. Written informed consent was obtained from each individual or patient.

## ELISA detection of serum sCLU

Serum sCLU protein was detected and calculated using an ELISA kit (Boster Biotechnology Company, Wuhan, China). The procedures were carried out according to the introduction of the manufacturer. All serum were stored at −80°C before measurements were taken, and both the standards and samples were run in triplicate. The OD450 was calculated by subtracting the background value, and standard curves were plotted using the CurveExpert 1.3 software program. Each sample was repeated at least 3 times.

## Statistical analysis

Statistical analyses were performed using SPSS.22 software. The t-test or the rank-sum test was used to evaluate the statistical differences. The Youden index, calculated as (sensitivity + specificity-1) was estimated to determine optimal cutoff values. The sensitivity and speciﬁcity were compared using receiver operating characteristic (ROC) curves. The associations between disease-free survival and overall survival and sCLU levels were estimated by the Kaplan–Meier, log-rank test, and Cox proportional hazard regression models. P < 0.05 was considered significant.

## Results

### Plasma levels of sCLU in IBC patients

The serum sCLU was assessed using ELISA in 1998 IBC patients,180 noncancerous patients and 170 healthy controls. The data showed that the serum sCLU protein levels were signiﬁcantly higher in patients with IBC (79.6 ± 11.5) μg/mL than those of the healthy controls (10.4 ± 4.8) μg/mL and noncancerous controls (19.5 ± 5.6) μg/mL (P < 0.0001, respectively), indicating that serum sCLU protein levels could be used as a biomarker for the IBC diagnosis.

## Serum sCLU levels and clinicopathological features in IBC patients

In the ﬁrst cohort of 1998 participants, sCLU level was higher in IBC patients with higher clinical stages (III)(92.6 μg/mL ± 13.8 μg/mL) and lymph metastasis (89.7 μg/mL ± 12.6 μg/mL) compared to the lower clinical stages (I+ II) (56.4 μg/mL ± 12.8 μg/mL) and without lymph metastasis (60.4 μg/mL ± 11.8 μg/mL) (Table 2, P < 0.0001, respectively). No relationship was found between levels of serum sCLU and age, tumor size, grade, distant metastasis, subtype, menopause, etc. (Table 2). These observations indicated that the progression of IBC is associated with increased serum sCLU levels.

**Table 2.** Relationship between sCLU levels and clinicopathological factors in 1998 IBC patients

Group	sCLU level (μg/mL)	P-value
Age (years)		0.089
<50	78.9 ± 11.6	
≥50	81.3 ± 11.7	
Tumor size		0.163
≤ 2 cm	77.7 ± 10.3	
>2 cm	80.4 ± 11.5	
Grade		0.251
G1-G1	78.3 ± 12.4	
G3	80.6 ± 11.8	
Clinical Stages		<0.0001
I–II	56.4 ± 12.8	
III	92.6 ± 13.8	
Lymph metastasis		<0.0001
Yes	89.7 ± 12.6	
No	60.4 ± 11.8	
Distant metastasis		0.173
Yes	82.6 ± 13.5	
No	78.4 ± 12.7	
Subtype		0.494
Luminal A	78.5 ± 12.7	
Luminal B	77.6 ± 13.1	
HER2 (+)	81.6 ± 13.6	
Triple negative	82.6 ± 12.7	
Menopause		0.560
Yes	79.0 ± 12.5	
No	79.8 ± 12.2	
ER		0.137
Positive(+)	81.6 ± 12.5	
Negative(-)	78.4 ± 12.5	
PR		0.382
Positive(+)	78.4 ± 11.8	
Negative(-)	80.9 ± 12.6	

**Figure 1.**
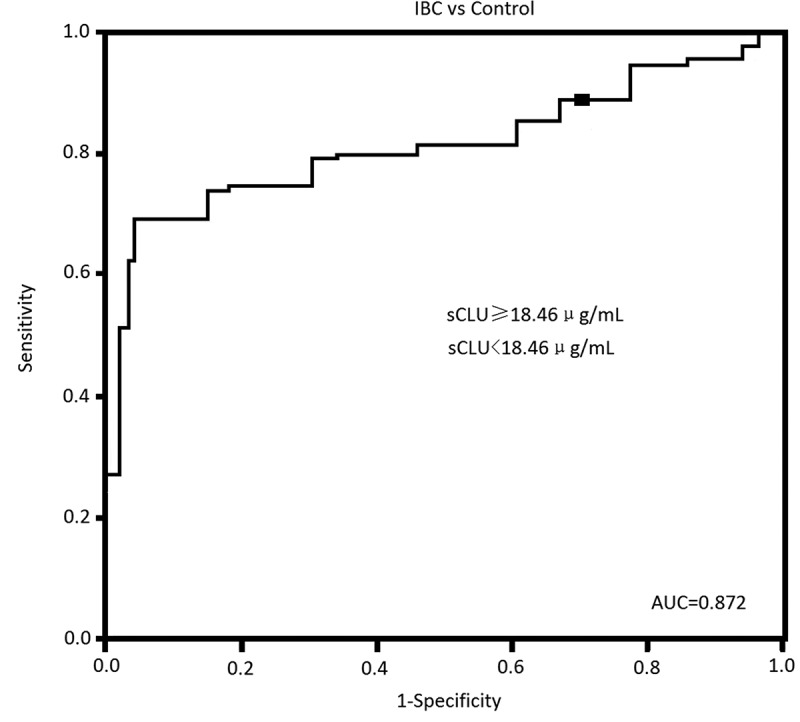
Serum sCLU levels in IBC and controls. Serum sCLU yielded an AUC value of 0.87 in distinguishing IBC from controls

**Figure 2.**
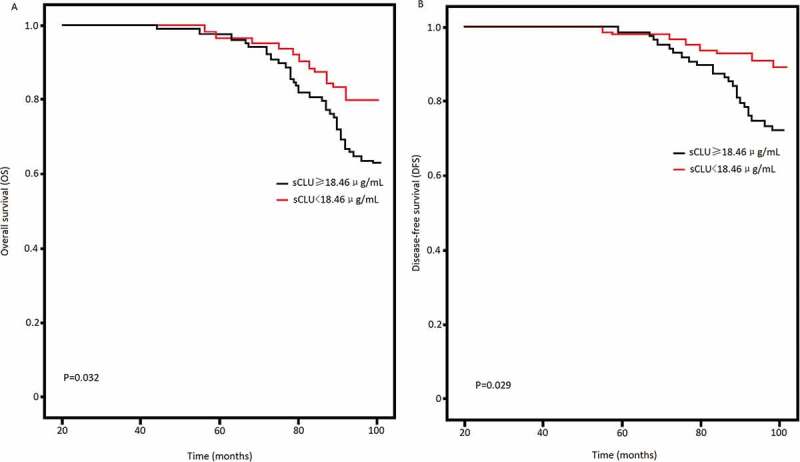
Kaplan-Meier survival curves. Percent survival rate was stratified by serum sCLU level. A, overall survival of patients;B, progression-free survival

We also found that serum sCLU protein levels >18.46 μg/mL would predict IBC patients with 86.26% sensitivity and 73.46% speciﬁcity. The AUC was 0.87 (Figure 1). Log-rank test of OS curves revealed that high serum sCLU levels were associated with the shorter OS (P = 0.032, Figure 2a) and DFS in IBC patients (P = 0.029, Figure 2b).

**Detection of serum sCLU levels in** newly diagnosed IBC patients

**Figure 3.**
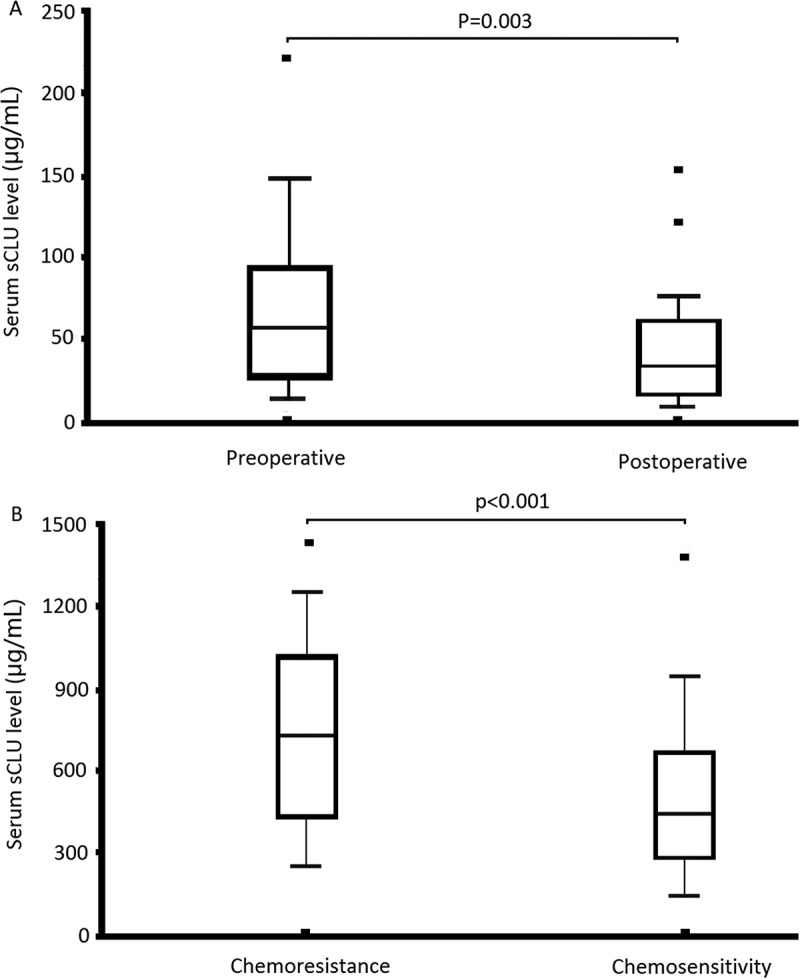
ELISA detection of serum sCLU levels. A, Surgical patients; B, Chemotherapy patients

Using cutoff value 18.46 μg/mL as the positive value for surgical patients, a statistically signiﬁcant association was observed between preoperative and postoperative serum sCLU levels (63.65 μg/mL vs. 35.14 μg/mL, P = 0.003; Figure 3a). In addition, serum sCLU levels were significantly higher in the chemoresistant patients than the chemosensitive patients (673.4 μg/mL vs. 292.7 μg/mL, p < 0.001, Figure 3 B).

## Discussion

In this study, we first detected the serum sCLU protein levels in IBC patients, noncancerous patients, and healthy controls and assess its diagnostic value and treatment responses in patients with IBC. The results showed that enhanced sCLU protein levels were found in patients with IBC than that in controls. Using 18.46 μg/mL as the cutoff value, the sCLU had a higher sensitivity and speciﬁcity in diagnosis of IBC, indicating that serum sCLU protein levels could be as a biomarker for the diagnosis of IBC.

Clusterin encodes a 449 amino acid precursor polypeptide which is cleaved to form the mature secreted clusterin protein which functions to scavenge and chaperone damaged proteins in the extracellular compartment of tissues [18,19]. Other functional variants of clusterin are generated by alternative splicing of the mRNA, producing a cytoplasmic variant, which can translocate to the nucleus and influence cell survival [19]. Clusterin is not detected in normal tissue, but overexpressed in many cancer tissues or cells [20–23]; therefore, the apparent ubiquitous presence of clusterin has the potential for it to be used as a biomarker for health and in disease. Previous reports have correlated clusterin expression with cell responses to stress [32], cell damage recovery [33], senescence [34], tumorigenesis and apoptosis [35]. Up-regulation of clusterin in breast cancers has been previously reported, but whether serum sCLU is enhanced still unknown. A previous study has reported that reduced sCLU might reflect poor outcomes of biliary atresia patients and have potential as a novel biomarker for the disease severity following Kasai-operation [36]. In human colon cancer, significant increase of sCLU in the serum of CRC patients as compared with controls; in addition, sCLU in blood showed a 55.6% sensitivity and 100% specificity [37]. In HCC and prostate Cancer patients, serum clusterin was significantly elevated, suggesting that serum sCLU is a potential novel serum marker for HCC and prostate Cancer [38,39]. In this report, we have demonstrated that serum sCLU levels were higher in IBC patients, especially in advanced IBC (stage III) and with lymph metastasis compared to the early-stage (stage I–II) and without lymph metastasis. Furthermore, the patients with higher sCLU levels have short survival than the patients with lower sCLU levels, suggesting that serum sCLU could be a useful prognostic marker in IBC. Expression patterns of clusterin at different stages indicate that this protein functions in the maintenance and/or progression of tumors, but it is not clear whether it plays a role in tumor establishment.

Elevated production of clusterin antigen, if secreted from tumor cells, may be detected in body fluids, such as serum. Therefore, serum sCLU could decrease after resection of tumors containing high expression of clusterin antigen. In our study, serum levels of sCLU protein showed a statistically signiﬁcant difference between preoperative and postoperative patients, and lower serum levels of sCLU protein were shown in the postoperative patients than the preoperative patients. These data indicated that serum levels of sCLU protein could predict the surgery responses of patients with IBC.

sCLU expression is documented to lead to broad-based resistance to anticancer agents and other unrelated chemotherapeutic agents, and targeted death-inducing molecules, tumor necrosis factor, Fas and TRAIL, or histone deacetylase inhibitors can also be mediated by sCLU [40]. Mechanistically sCLU could mediate multidrug resistance to a broad range of unrelated chemotherapeutic agents. sCLU is also readily induced by therapeutic agents and confers survival advantage to cancer cells [41]. Therefore, sCLU ablation via specific antisense oligonucleotides as a means to chemosensitization toward clinically established drugs could be a potent strategy to overcome drug resistance. Serum sCLU levels could be effectively reduced with effective chemotherapeutic agents [42]. In the study, we found that serum sCLU protein was higher in the chemoresistant patients compared to the chemosensitive patients. These data demonstrated that serum sCLU levels could predict the chemotherapy responses in patients with IBC. However, some limitations exist in the study that some clinicopathologic data were not enough.

## Conclusion

The study aimed to investigate the relationship between serum secreted clusterin (sCLU) protein and clinicopathological characteristics and chemotherapy sensitivity evaluation in IBC. It was demonstrated that the serum sCLU protein level was signiﬁcantly higher in IBC patients, and strongly correlated with higher clinical tumor stage, lymph node metastasis, shorter overall survival and disease-free survival (DFS). Serum sCLU protein was also higher in the chemoresistant IBC patients. Therefore, detection of serum of sCLU could help to predict prognosis and monitor the treatment responses. However, there are a number of challenges to overcome in order to successfully translate these promising laboratory results into efficacious therapies in clinical practice.

## Supplementary Material

Supplemental Material

## Highlights

1. Serum sCLU level was higher in patients with invasive breast cancer
2. Higher serum sCLU level was related to high tumor stage and lymph metastasis
3. Lower serum sCLU level was observed in the postoperative patients
4. Higher serum sCLU level was observed in the chemoresistant patients
