# Supplementary figures and images for: Clinical importance of serum secreted clusterin in predicting invasive breast cancer and treatment responses
Source: Bioengineered. 2021 Jan 28;12(1):278–85. doi: 10.1080/21655979.2020.1868732 (PMC8806267; doi:10.1080/21655979.2020.1868732)

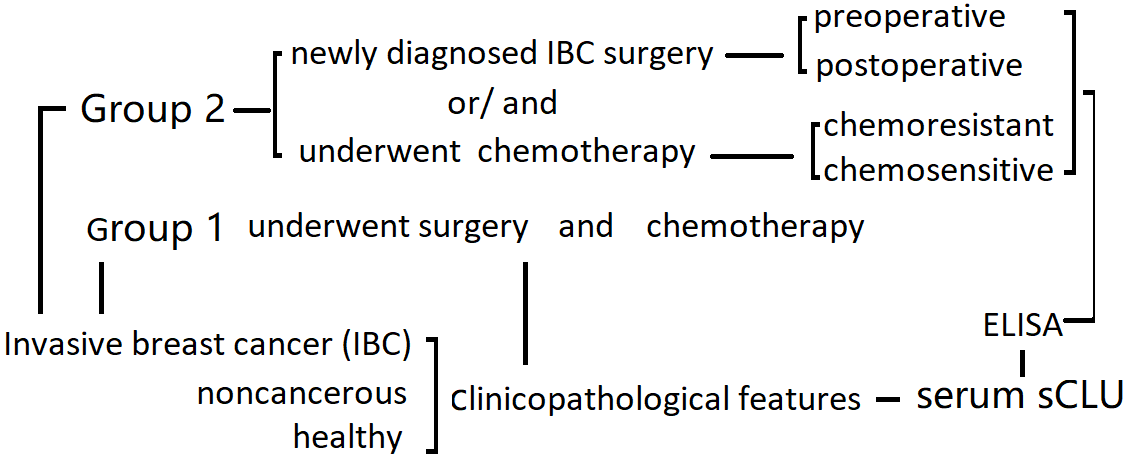

Supplement: Supplemental Material [file KBIE_A_1868732_SM3263.tif]
